# Mucinous Cystic Neoplasm of Mesentery: A Case Report

**DOI:** 10.7759/cureus.21482

**Published:** 2022-01-21

**Authors:** Jayaraghavan R, Ganesh Bhat

**Affiliations:** 1 General Surgery, Byramjee Jeejeebhoy Government Medical College (BJGMC) and Sassoon General Hospital, Pune, IND; 2 Surgery, Baramati Hospital, Pune, IND

**Keywords:** mesenteric cyst, rare, benign, abdominal mass, cyst, mesentery

## Abstract

Mucinous cystic neoplasms are very rare tumours. They may originate from ovaries, pancreas or other intra-abdominal sites but they rarely originate from the mesentery. They can be asymptomatic or present as an abdominal mass or abdominal pain. We present the case of a 28-year-old woman who presented with epigastric pain and cystic mass per abdomen with a diagnosis of mesenteric cyst made on further imaging studies. Subsequent excision and histopathological analysis demonstrated the cyst to be a mucinous tumour arising from the mesocolon. Mesenteric cyst must be considered as one of the differentials in abdominal cystic lesions.

## Introduction

Mucinous cystic neoplasms (MCN) can originate from the ovary or from other extraovarian sites including the pancreas, liver, kidneys, appendix and mesentery. Incidence of MCN is about 1:2,50,000 [1]. Mesenteric neoplasms are classified into three types i.e., benign cystadenomas, borderline, and invasive carcinomas according to histopathological features [2]. Mucinous cystic neoplasms of the mesocolon are very rarely found [2].

## Case presentation

**Figure 1:**
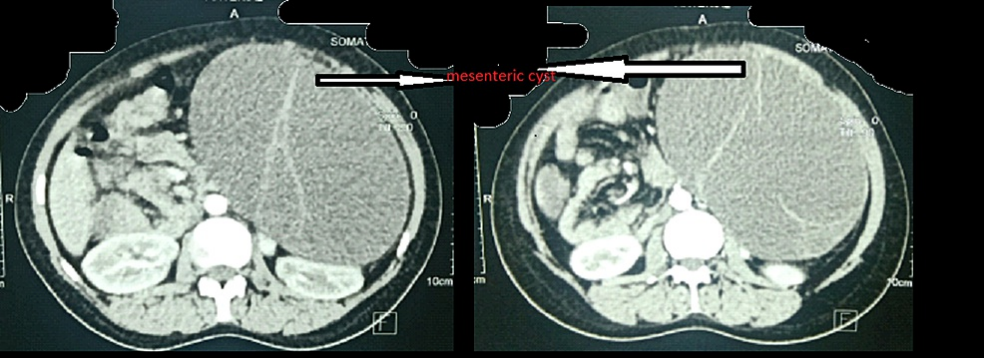
CT image of the abdomen The CT scan reveals a large, well defined multiloculated thin-walled cystic lesion with thin incomplete enhancing septae.

A 28-year-old female presented to the surgical outpatient department (OPD) with the primary complaint of epigastric pain for two months that had progressively increased with sitting for prolonged periods and relieved on lying down, and no history of trauma. The patient had a similar episode six years prior when she was diagnosed with a mesenteric cyst and an ultrasonographic guided aspiration was done. Cytology report revealed the presence of inflammatory cells predominantly macrophages with the absence of any neoplastic cells. On physical examination her vital parameters were stable. On abdominal examination, the following was observed: soft elastic abdomen with soft cystic fluctuant 18 cm x 15 cm mass palpable in left hypochondriac, epigastric and umbilical region. Ultrasonography of the abdomen revealed heterogenous hypoechoic cystic lesion measuring 19.8 x 15.6 x 18 cm with minimal vascularity with echogenic septae and debris within the pancreatic tail region with the displacement of kidney and spleen posterolaterally. Differential diagnoses that can be considered are pancreatic pseudocyst and mesenteric cyst. The CT scan (Figure 1) revealed a large well defined multiloculated thin-walled cystic lesion with thin incomplete enhancing septae intraperitoneally 16.3 x 10.8 x 17.2 cm abutting the left kidney and spleen with a maintained fat plane. Anterolaterally it was abutting the abdominal wall. The pancreas and spleen were normal. Possible differentials are mesenteric cyst and lymphangioma. 

**Figure 2:**
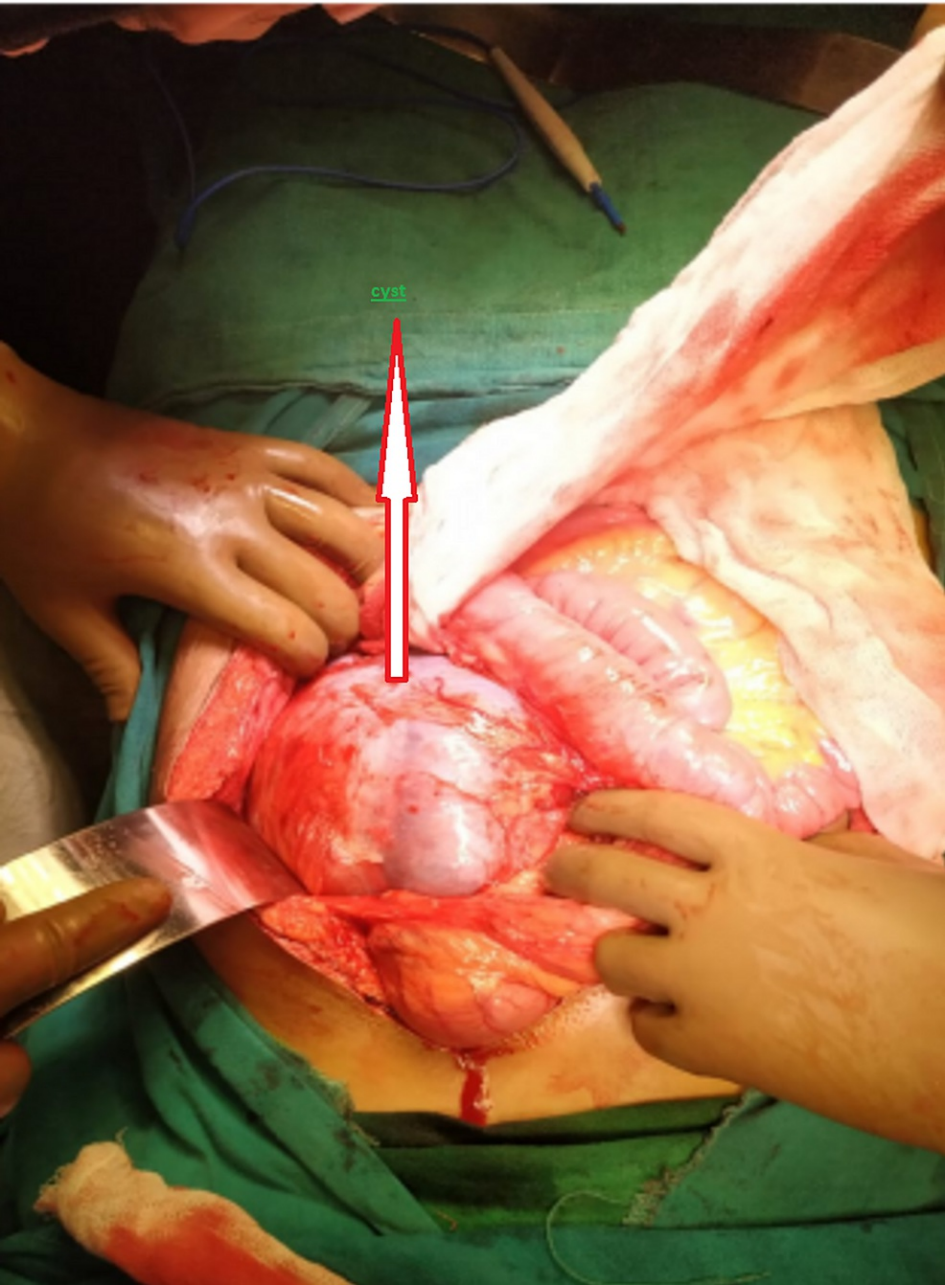
Intraoperative image A large cystic lesion can be seen arising from the transverse mesocolon.

Intraoperative findings revealed an 18 x 14 x 12 cm cyst superior to the transverse colon displacing spleen posterolaterally arising from the mesentery of the transverse colon (Figure 2). The cyst was dissected and the pedicle arising from the mesentery of the transverse colon was ligated and cut. Enucleation of the cyst was done. Subsequent postoperative recovery was uneventful.

**Figure 3:**
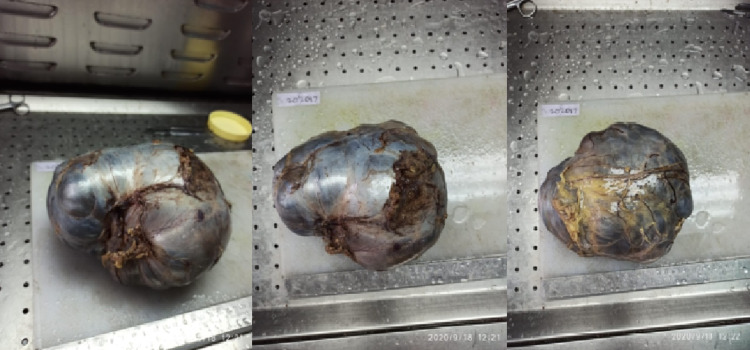
Gross specimen The above images show a large, bluish-black globular mass with a smooth surface and intact capsule.

On gross examination, a large globular mass 18 x 14 x 12 cm bluish-black in colour, was observed with congested blood vessels and was tense and globular with the capsule intact and smooth (Figure 3). The mass weighed 1700gm. 

**Figure 4:**
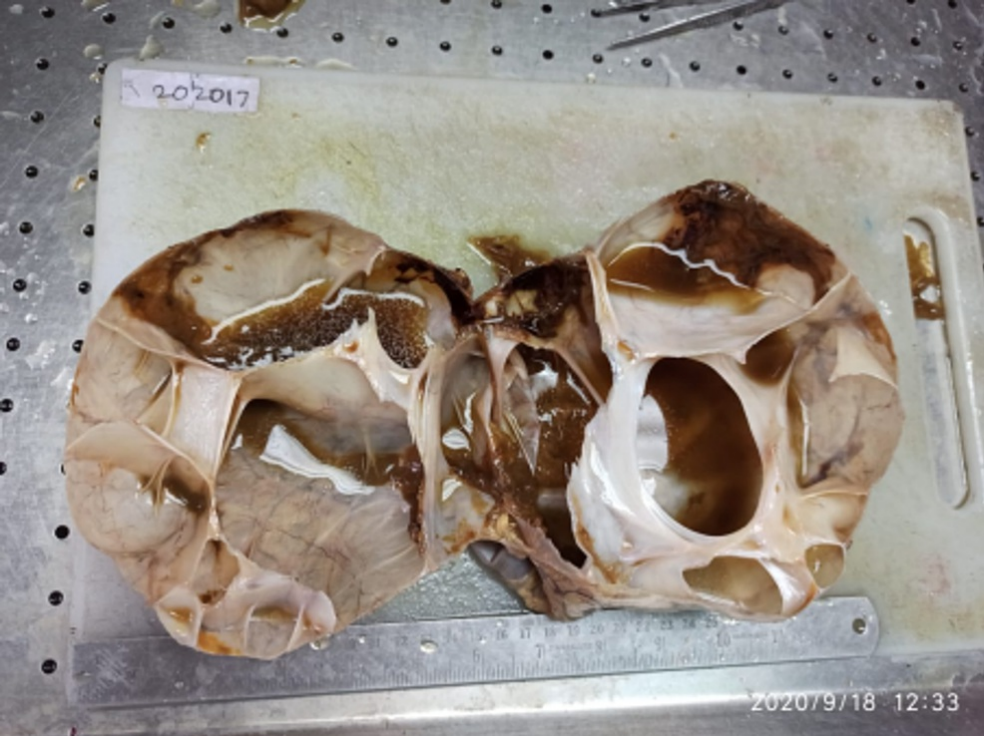
Cut specimen The cut surface shows a multiloculated cyst exuding yellowish-green fluid.

On cutting open, the cyst exuded a greenish-yellow fluid (Figure 4). At the location of the cut, the lesion appeared multiloculated. The inner surface appeared smooth, and no solid areas were identified. Mucous was identified in certain places. Cyst walls were whitish and thin. 

Multiple sections studied from the cyst show a fibromuscular cyst lined by cuboidal to columnar mucin secreting epithelium. Also seen were a few mucin secreting glands in the wall. No pleomorphism, no nuclear stratification and no stromal invasion. The impression was that of a mucinous cystadenoma.

The patient was followed up after three and six months, respectively, with no complaints and the ultrasound of the abdomen showed the complete disappearance of the cyst.

## Discussion

Mucinous cystic neoplasm of the mesocolon is a very rare lesion. Mucinous cystic neoplasms arise in ovarian as well as extraovarian sites. The cyst wall is lined by mucin-secreting flat, cuboidal and/or columnar epithelium. An extra-ovarian MCN can be due to an implanted or ectopic ovarian tissue, supernumerary ovaries or, monolinear development of a component of teratoma [3]. Extraovarian MCN can be due to coelomic mucinous metaplasia of epithelial cells or invaginated peritoneum along the course of ovarian descent [4]. These cysts can have various complications like infection, haemorrhage, and malignancy. The malignancy rate in these cysts is 3% [3]. There are no definitive diagnostic criteria but radiology can confirm the site of origin of cysts, which can help plan surgery. Only postoperative histopathology can differentiate between various histopathological grades. The mainstay of treatment is surgical excision. There is no role of conservative management as there is a chance of malignancy and rupture of the cyst.

## Conclusions

Mucinous cystic neoplasm of the mesentery is a rare condition presenting mostly in women of reproductive age. It is usually found incidentally but may present with symptoms similar to a mesenteric cyst. They are difficult to diagnose accurately on imaging. A CT scan can pinpoint the site of origin and structures it compresses. Aspiration cytology has no value as it has high false negative value. Aspiration is not a preffered mode of treatment as we may not be able to tell histopathological diagnosis and grade. Moreover, it may lead to recurrence as in this case. There is also the risk of malignant transformation. So, surgical excision is the mainstay of treatment. It should therefore be concluded that these cysts should be treated by complete excision rather than drainage because of risk of missing the diagnosis of MCN, recurrence and malignant transformation. 
